# "Cramming" in Phthisis

**Published:** 1901-06-22

**Authors:** 


### "CRAMMING" IN PHTHISIS.

At the same meeting a very important communication was made by Dr. F. W. Goodbody, Dr. Noel D. Bardsley, and Dr. J. E. Chapman, who had made some very elaborate investigations as to the metabolism in phthisis with the object of ascertaining the limit of utility of over-feeding in that disease. Special efforts had been made to ascertain what number of calories (in the food) per kilogramme of body-weight was the most suitable in this disease, as this was the easiest way of determining a suitable diet, and one likely to give better results than mere "cramming," the strain of which on the organism must be very excessive. The researches were made at the Brompton Hospital upon cases selected by Dr. Kingston Fowler, and the estimations were made at the laboratory of University College. It was found in the first instance that healthy persons could not stand excessive diet as well as those who were suffering from pulmonary tuberculosis. Even although they might increase in weight digestive disturbances were produced from which they did not recover for two or three weeks. Among consumptives, also, the cases did not progress so well under excessive feeding, even though they might be gaining flesh. It is clear then that some guide other than the power of taking food is to be sought in regard to the degree to which over-feeding should be carried in the treatment of phthisis. Among the conclusions arrived at by the authors were, that both clinically and experimentally tuberculous patients showed very satisfactory results on their diet being increased slightly above the original, showing that the state of the appetite gives too low an estimate of the requirements of the system; that comparatively large diets were well borne by those who were much below their normal weights, but were not so well supported—nor were such good results obtained in—patients up to their normal weight and with arrested disease; that in all cases very large diets gave unsatisfactory results, the gain in weight being at the expense of the general health, as shown by the failure of appetite, marked digestive disturbance, and increased intestinal putrefaction; that moderately large diets gave the best results, and could probably have been continued for an indefinite period; that the patients complained least of discomfort on the diets which gave the best results experimentally, and that the onset of severe dyspeptic trouble coincided with deterioration in the experimental results. The investigators found that the number of calories per kilogramme of body weight which gave the best results was between 50 and 70, and that in the most advanced cases under their observation the most suitable diet was one consisting of 129·87 grammes proteid, 135·05 fats, and 225·56 carbohydrates. From a practical point of view we deduce the conclusion that the more a patient has gone down from his normal weight, the more useful will be overfeeding, but that its utility is seen in all cases; that the overfeeding which is most appropriate is something distinctly in excess of what mere appetite would guide a patient to take, but something distinctly below what will produce stomach or bowel trouble; and thus that the "forced feeding" of which we hear so much, in contradistinction to mere overfeeding as just defined, is a thing to deprecate. The same old advice which is applicable in so many other circumstances may then be given also in regard to the dietetic treatment of consumption—*in medio tutissimus ibis*.

